# Supplementary material for: Are carbon emissions trading and green financial instruments synergistic? -Comprehensive quantitative research based on content analysis
Source: PLoS One. 2024 Mar 7;19(3):e0298601. doi: 10.1371/journal.pone.0298601 (PMC10919601; doi:10.1371/journal.pone.0298601)
Supplement: S1 Data — (ZIP) [file pone.0298601.s001.zip › Data/地级市Z_F工作报告.zip!/╣π╢1⁄2/╓Θ║ú╩╨/└┤╫╘╣1⁄2╓┌║┼í╛┬φ┐╦╩2╛▌═°í┐_2009.docx]

各位代表：

　　现在，我代表市人民政府向大会作政府工作报告，请予审议，并请政协各位委员和其他列席人员提出意见。

　　回顾与总结

　　2008年，是我市经受严峻挑战和考验、经济社会保持平稳较快发展的一年。面对国内外环境的深刻变化，尤其是遭遇历史罕见的国际金融危机的冲击和两次强台风、年初雨雪冰冻灾害的影响，在市委的正确领导下，在市人大及其常委会和市政协的监督支持下，市政府认真贯彻落实省委十届二次、三次全会和市委六届三次、四次全会精神，紧紧围绕“建设生态文明新特区、争创科学发展示范市”的目标，迎难而上，扎实工作，取得了来之不易的成绩。

　　——经济保持平稳增长。全市实现生产总值992.1亿元，同比增长9％。人均生产总值67591元，增长7.8％。财税总收入318亿元，增长27.7％。地方财政一般预算收入92.3亿元，增长21.8％。人均财政一般预算收入居全省第二位，达到6233元，增长19.6％。进出口总额468.4亿美元，增长17.5％，增速居珠三角第二位。固定资产投资372.3亿元，增长7.9％。社会消费品零售总额359.7亿元，增长19.3％。金融机构各项存款和贷款余额分别比年初增长13.5％、12.4％。全年物价上涨4.6％，走势平稳回落。

　　——重大项目实现新突破。重大项目取得突破性进展，与中国船舶工业集团、中国航空工业集团分别签订《关于推进中船华南大型造船项目建设的框架协议》和《关于发展航空产业的战略合作协议》，共同在珠海建设海洋工程装备制造和通用飞机制造基地。有7个项目列入省重点建设项目，计划总投资额1147.7亿元。总投资540亿元的16个重大项目动工建设，年度投资已达到102亿元。涵盖基础设施、现代产业、民生工程等多个领域、总投资约220亿元的一批重大项目，在去年底集中动工、竣工。新引进项目508个，其中投资总额一千万美元以上的外资项目19个，合同投资总额15.6亿美元。

　　——产业发展不断优化。产业结构调整势头良好，第三产业所占比重由上年的41.9％提高到42.4％。新增省产业集群升级示范区两家。实现工业总产值2668亿元，增长7.8％，其中，高新技术产品产值突破一千亿元，占全市工业总产值的39％。工业增加值509.4亿元，增长8.9％。产学研结合取得新进展，4家国家重点实验室落户珠海，产学研示范基地和科技创新公共实验室增加到20家，建成我市首家国家级企业技术中心。

　　——区域合作不断深化。珠港澳台合作取得可喜成果。引进港澳项目247个，合同外资9.8亿美元，其中服务业项目183个。全年港澳贸易总额99.9亿美元，增长11.6％。成立了珠澳合作专责小组。第三条供澳原水管建成通水，对澳日供水能力达到50万吨，概算总投资近10亿元的竹银水源工程动工兴建。对澳供电电压等级提高到220千伏。全省首个台湾农民创业园落户金湾。珠港合作经营管理的珠海机场年旅客吞吐量达到112万人次，增长8％，高于全国平均水平4.7个百分点；运输飞行1.1万架次，增长17％。

　　——民生事业取得新进步。财政一般预算用于社保、就业、教育、医疗卫生等民生支出42.1亿元，增长24.6％。城乡居民生产生活条件不断改善。城镇居民人均可支配收入20949元，增长8.6％；农渔民人均纯收入8048元，增长5.6％。居民储蓄存款728亿元，人均存款增加8833元。全市建成市政设施项目65个，市疾病预防控制西部中心、社会福利中心和104个农村卫生服务中心竣工投入使用。发放低保金和租赁住房补贴2472万元，新推出廉租房300套，累计改造农场危房2312户。新增就业岗位55198个，完成省下达任务的138％，城镇登记失业率为2.79％。对先天性心脏病儿童、白内障老人、精神病人、孤儿百分百实施救治救助。率先推行新型农村合作医疗向城乡居民基本医疗保险并轨，农民和被征地农民医疗保险参保率达98.2％。农村养老保险老年津贴标准提高50％。基本社会保险参保总人数331万人次，覆盖率为91.5％。

　　——城市品牌继续提升。宜居城市建设取得新进展，城市环境质量保持优良，环保责任考核保持全省第一，污染减排和江河水整治考核连续多年位居全省前列。市容整治和城管执法力度加大，全年查处违法行为32181宗。出台了农贸市场管理体制改革方案。已建成3个“全国环境优美乡镇”、67个省级和市级生态示范区。我市入选“全国生态文明建设试点地区”，被评为“全国创建文明城市工作先进城市”和“国家优秀创新型城市”。三灶镇荣获“全国文明村镇”称号，白蕉镇、井岸镇坭湾村荣获“全国创建文明村镇工作先进村镇”称号，珠海移动公司、市邮政局荣获“全国文明单位”称号，市烟草专卖局、香洲区拱北街道办事处荣获“全国精神文明建设工作先进单位”称号，全市文明程度不断提高。

　　——对口帮扶扎实推进。大力支援地震灾区抗震救灾和恢复重建工作，募集款物2.25亿元，市财政安排援建资金7450万元，其中绵虒镇小学和村道维护一期工程建设已投入资金3500万元。对重庆市巫山、巫溪、奉节三县和揭阳市揭西县投入财政帮扶资金1290万元。

　　一年来，我们切实抓了以下几个方面的工作：

　　一是采取积极有效措施，应对金融危机保增长。把积极应对国际金融危机、帮助企业渡过难关作为政府工作重中之重。加强经济运行监测和分析，及时研究出台了有关外经贸、招商引资、现代服务业、民营及中小企业、促进就业和稳定生产等10多个文件，推出一系列政策措施，刺激经济增长。加大扶持企业力度，安排3.6亿元资金支持企业开拓国内市场、融资贷款和科研创新。推广“四位一体”融资模式，20家银行和担保机构加入融资推广工作。及时建立应对金融危机和项目协调联席会议制度，妥善处理企业裁员、欠薪逃匿等问题。清理收费项目，实施部分收费缓征或免征政策，仅连续两次调低失业和工伤保险缴费比例，就为企业减负2亿多元。开展“五问三送”活动，现场协调解决企业发展和项目建设中的问题。在应对金融危机中，把拉动经济增长与改善民生结合起来，把优化发展环境与强化社会保障结合起来，把克服当前困难与促进长远发展结合起来，促进经济平稳较快增长。

　　二是推进交通等重大项目建设，着力突破发展瓶颈。坚持把重大项目建设作为加快经济发展的强劲动力。科学编制重大项目建设计划，加强综合协调，完善管理和考核机制。落实交通项目融资90亿元，追收土地出让欠款6.4亿元，积极引进战略投资者，拓宽了项目融资渠道，为重大项目建设提供资金保障。西部沿海高速珠海段支线、南湾立交、湖心路口至机场段省道改造工程竣工通车。首个5万吨级集装箱码头泊位建成投产。一批重大项目加紧推进，广珠铁路珠海段征地工作全部完成并全面动工，广珠城际轨道交通工程累计完成总投资超过六成，机场高速、高栏港高速公路软基工程动工建设，港珠澳大桥主体工程建设可望在2009年内动工。茂盛海洋石油、格力压缩机二期、凯邦电机、PTA二期等项目建成投产，500千伏国安输变电工程加快建设。

　　三是加大园区整合和招商引资力度，优化经济结构和布局。把加快现代产业体系建设作为优化经济结构的重要途径。整合园区资源，促进产业集群发展，形成了新的园区布局。航空产业园建设顺利推进。围绕城市和产业发展，编制了14项专项规划，清理处置闲置工业用地1634.6万平方米。完善招商引资激励机制，加大招商引资力度。全年实际吸收外商直接投资11.4亿美元，增长11％；新批境外投资和增资企业20家，增长150％。引进内资注册资本总额30.1亿元，增长37.2％。大力发展现代服务业，成功举办第七届中国航展和旅游年等系列活动，出台促进房地产业发展政策措施，制定“退二补公进三”鼓励政策，优化城市规划布局。大力发展现代农业、城郊型农业、出口型农业，铭海投资公司成为我市首家国家级农业龙头企业，新增5家省级和市级重点农业龙头企业，白蕉镇、莲洲镇国家级农业示范区加紧建设，农业产业化水平有了新的提高。实现农业总产值52.5亿元，增长1.8％。分别与揭阳市、茂名市签署协议，合作建设产业转移园。

　　四是推动体制改革和珠港澳合作，深入推进改革开放。坚持以改革开放为强大动力，增创发展新优势。稳步推进体制改革，成立港口管理局，组建市政园林局，横琴实行“区镇合一”新体制。推进公共资源市场化配置，深化国有资本运营体制和产权制度改革，实施城市公交和污水处理特许经营改革，组建了珠海港控股集团和交通集团。珠海市商业银行改革重组步伐加快。经营性国有企业实现利润总额39.8亿元，增长33％。外经贸和口岸工作取得新成绩，获得省政府授予的吸收外商直接投资、口岸大通关建设等8个一等奖和两个二等奖。口岸发展规划获省批准，九洲港口岸改造基本完成，西域、恒基达鑫码头对外开放。全年经珠海口岸出入境旅客8833.7万人次，增长4.7％。港口集装箱吞吐量65.5万标准箱，增长3.8％。积极推进对港澳服务业开放先行先试工作，商务部批准珠海作为首批落实CEPA示范城市。

　　五是切实改善民生，大力加强社会建设。悠悠万事，民生为大。我们十分注重加快推进以改善民生为重点的社会建设。出台《珠海市关于促进民办教育发展的若干意见》和《珠海市关于促进学前教育发展的若干意见》，新建一批中小学校，采取初高中分离办学等新举措，促进义务教育均衡发展。高中阶段毛入学率提高了2.6个百分点，达到97.8％。普通高考取得优异成绩。大学园区稳步发展，全日制在校生达8.5万人，省部产学研结合示范市建设稳步推进。重大文化项目取得新进展，古元美术馆和图书馆新馆投入使用。在全省率先通过档案事业发展综合评估。“斗门水上婚嫁习俗”列入国家非物质文化遗产保护名录。出台《关于构建新型公共卫生体系的实施意见》，推行全民基本医疗保障制度。出台《关于进一步加强外来务工人员服务工作的若干意见》，切实维护外来务工人员合法权益。出台《规范我市农民（被征地农民）建房管理的若干意见》，从制度上解决了长期困扰农村发展的村民报建问题。乾务水厂、井岸污水处理厂建成运营。成功举办庆祝改革开放30周年和第七届市运会等活动。我市5名运动员参加北京奥运会并取得良好成绩。双拥共建、国防建设和民兵预备役工作扎实推进，我市连续第七次获得全省“双拥模范城”称号。连续第20年完成省下达的人口计划控制任务。香洲区成为全国社会治安综合治理先进集体和残疾人社区康复示范区，金湾区进入“全国计划生育优质服务先进单位”行列，斗门区成为“国家食品安全示范区”。推进行业场所“三严”整治，从源头上预防和化解社会矛盾，在全省率先建立人民调解、行政调解与司法调解有机衔接的大调解机制。我市被评为全省禁毒综合考评先进单位，成功创建全国科技强警示范市。强化各级信访责任制，狠抓各项维稳措施的落实，妥善处置重大公共突发事件，确保社会稳定。安全生产形势平稳，火灾事故连续两年“零死亡”。有效组织抗击“鹦鹉”、“黑格比”强台风袭击，市政府投入1.1亿元及时安置受灾群众和恢复生产，努力把灾害损失降到最低程度。

　　六是切实抓好制度建设，不断提高行政效能。始终把政府自身建设作为转变职能、优化政务环境、服务市民的重要抓手。把制度建设和监督作为依法行政的重要内容，全年提请市人大常委会审议地方性法规草案5件，已颁布3件。颁布政府规章和规范性文件34件。完善市政府常务会议议事规则，规范政府决策行为。深入开展“行政效能问责年”和政风行风评议活动，加强行政监察和审计监督，廉政建设和效能建设取得实效。30个政府部门发布了责任白皮书。启用电子公文协同处理和建设项目协同审批系统，进驻市行政服务中心审批事项办结率达99％、提前办结率超过50％，建设工程报建审批效率提速约三分之一。实行涉车缴费集中办理，为市民办事提供了方便。自觉接受市人大和市政协监督，充分发挥各民主党派、工商联、人民团体和无党派人士的作用。提请市人大常委会审议各项工作报告29项，办理市人大议案5件、市人大代表建议176件、市政协提案401件。

　　一年来，中央和省驻珠各单位、驻珠军警部队在推动我市经济社会发展中发挥了重要作用。新闻出版、民族宗教、统计、外事、侨务、海防、打私、打假、人防、气象、仲裁、地方志、港澳流动渔民等工作取得新进步，老龄、福利、红十字、慈善和残疾人事业健康发展。

　　各位代表，过去一年，在严峻的挑战和考验面前，全市人民团结拼搏、奋发有为、共克时艰，向改革开放30周年和珠海经济特区成立28周年交出了新的答卷。能够取得这样好的成绩，靠的是党中央、国务院和省委、省政府的正确领导，靠的是历届领导班子团结带领全市人民努力拼搏、艰苦创业打下的良好基础，靠的是社会各界的大力支持。在此，我代表市人民政府，向各位人大代表、政协委员和各民主党派、工商联，向奋斗在各条战线的全市广大干部群众，向中央和省驻珠单位、驻珠军警部队官兵，向为珠海发展作出奉献的老领导、老同志，向所有关心和支持珠海发展的港澳同胞、台湾同胞、海外侨胞、国际友人和社会各界人士，表示崇高的敬意和衷心的感谢！

　　回顾过去的一年，在看到成绩的同时，我们也清醒地认识到，我市在发展中还存在一些不容忽视的问题：一是经济结构性矛盾日渐显现。需求不足导致企业订单减少，使外向度高的我市经济受到较大冲击。同时，由于大型企业不多，服务业和民营经济比重不高，自主创新能力不够强，企业生产经营面临很大困难，经济增速出现较大回落。二是财政支出尤其是政府投资项目资金压力前所未有。去年市政府投资计划中有91.75亿元在土地出让收入中安排，占政府投资计划的78.3％。受国际金融危机和调控政策的影响，为确保土地资源保值增值，促进我市房地产市场健康有序发展，市政府决定暂不出让商用土地，因此造成了部分政府投资项目资金无法到位。三是城乡差距仍然较大。统筹城乡发展是今后一个时期的重要任务。特别是西部地区防灾减灾设施欠账较多，水利设施达标任务艰巨，保持农业稳定增长、农民持续增收难度加大。农村公共服务水平不高，西部大开发步伐有待加快。四是政府服务与群众期望仍然存在较大差距。政府自身建设有待加强，行政管理仍然存在薄弱环节，一些涉及群众利益的问题还没有得到较好解决，社会管理和公共服务职能有待强化；形式主义、官僚主义、推诿扯皮等现象依然存在，“事难办”的问题还没有得到根本解决。针对这些问题，我们将采取有效措施，切实加以解决。

　　形势与任务

　　2009年，将是新世纪以来经济发展最为困难的一年，也是各种机遇与挑战并存的一年。

　　目前，国际经济环境依然复杂多变，国际金融危机尚未见底，对实体经济的影响还将进一步加深，经济下行压力加大，经济发展面临严峻形势。从我市情况看，外经贸环境面临多重挑战，外向型经济发展尤其是外贸出口和吸收外资将面临巨大的困难。在全国出口和实际吸收外资持续下滑的大背景下，今年1月份我市部分重要经济指标也出现负增长，经济增速回落的趋势还没有改变。估计今年是我市实现经济社会发展预期目标最为困难的一年。《珠江三角洲地区改革发展规划纲要》（简称《纲要》）的实施，即使我们看到美好前景，同时，也感到任务繁重。特别是建设珠江口西岸核心城市和交通枢纽城市，要求迫切，但又需要一个过程，不是一年两年就可以建成的，还有许多工作要做，责任很重。今年财政减收增支因素增多，土地出让形势也不容乐观。为落实好扩内需、保增长政策措施，必须千方百计筹措资金，满足项目建设对资金的巨大需求，确保政府投资计划落实。新的情况、新的形势不仅要求政府在依法行政、公共服务、社会管理等方面必须适应发展，而且又对政府的决策力、执行力、公信力提出了新的更高的要求。因此，今年的工作将面临更大的困难，承受更大的压力。

　　面对当前的严峻挑战，必须做好“打硬仗”的准备，要充分估计困难，周密部署工作，坚决推动落实，努力化传统发展模式之“危”为科学发展模式之“机”。尤其要牢牢把握和利用好当前的有利因素：一是《纲要》从广东乃至国家发展全局的高度，要求珠三角地区“科学发展、先行先试”，赋予了珠三角地区发展更大的自主权，支持率先探索科学发展的新途径、新举措，赋予继续承担改革“试验田”的历史使命，同时也明确了我市的发展定位、发展目标和战略重点，给我市发展提供了新的机遇和强大动力。二是国家实施积极的财政政策和适度宽松的货币政策，特别是一系列保增长、扩内需、调结构的政策措施，不仅有利于推动全国、全省经济平稳较快发展，而且也有利于我们筹措资金，加快交通等重大项目建设。中央高度重视粤港澳合作，近期推出了建立珠三角世界级大都会、推动重点基建项目等7个方面14项措施支持港澳经济发展，这些都将为加强珠港澳合作提供新的发展空间。特别是下一步横琴新区建设，必将成为我市新一轮发展的亮点。三是珠海具备特区品牌、城市区位、生态环境、发展空间和科教人文等比较优势，这些优势已成为我市未来的巨大发展潜力。当前，国际产业向亚太地区转移的趋势没有变，中国经济总体向好的基本态势没有变，为我市聚集外资、引进项目，承接高端制造业、高新技术产业和现代服务业转移，为提高自主创新能力，推动产业优化升级，转变经济发展方式，提供了千载难逢的条件。四是我市在改革开放中积累了比较坚实的物质基础，形成了具有活力的体制机制，经济实力、区域竞争力和抵御风险能力大大增强。解放思想学习讨论活动进一步凝聚了共识，调动了各方积极性，形成了良好发展氛围。五是一代又一代特区开拓者、领导者和建设者牢记使命，大胆探索、开拓创新，形成了敢闯敢试、敢为人先的特区精神，创造了特区建设的宝贵经验。这些都为我们干事创业提供了有利条件和广阔空间，也是我市保持经济平稳较快发展的动力所在和重要支撑。

　　各位代表，越是困难的时候，越要统一思想，坚定信心，凝聚合力；越是困难的时候，越要头脑清醒，认清形势，攻坚克难；越是困难的时候，越要弘扬特区精神，敢想敢干，破解难题。只要我们同心同德、群策群力，坚毅自信、奋发进取，就一定能够化压力为动力，化挑战为机遇，开创政府工作新局面！

　　今年我市经济社会发展的主要预期目标是：生产总值增长8.5％，力争人均生产总值同步增长；工业增加值增长11.5％；固定资产投资增长15％；社会消费品零售总额增长15％；居民消费价格总水平涨幅控制在2％以内；外贸进出口、实际吸收外商直接投资额力争有所增长；引进内资注册资本金增长20％；财政一般预算收入力争增长6％；单位生产总值能耗下降3.1％以上；二氧化硫排放量、化学需氧量分别控制在3.51万吨和2.89万吨以内；城镇居民人均可支配收入和农渔民人均纯收入分别增长6％和6.5％；城镇登记失业率控制在3.5％以内；户籍人口自然增长率控制在8‰以内。

　　思路与举措

　　今年市政府工作的总体思路是：深入贯彻落实科学发展观，围绕建设生态文明新特区、争创科学发展示范市的目标，全面贯彻省委十届四次全会和市委六届四次、五次全会精神，以深化改革开放为动力，以实施《珠江三角洲地区改革发展规划纲要》为主线，按照“三促进一保持”的要求，认真落实市委确定的“五大任务”，着力构建交通、产业、城市三大格局，切实扩大内需保增长，切实转变发展方式，切实优化发展环境，努力在应对金融危机、构建现代产业体系、重大项目建设、改善民生福利、创新体制机制等方面取得新突破，确保完成全年各项工作任务。

　　今年要着力抓好以下工作：

　　一是以建设珠江口西岸核心城市为重点，着力推进《纲要》的实施。

　　全面履行《纲要》赋予珠海的新使命。《纲要》的实施，对珠海未来发展意义重大。《纲要》明确了珠海作为区域中心城市、珠江口西岸核心城市和交通枢纽城市的定位，这是中央和省对珠海当前和未来的发展要求，也是我市建设生态文明新特区、争创科学发展示范市的重要内容。要深刻领会、全面把握《纲要》的编制背景、重要意义、总体要求、发展目标和具体内容。要紧紧围绕目标定位，在不断提高基础设施辐射能力、产业集聚能力、城市综合服务能力等方面狠下功夫，加快经济发展，完善城市功能，努力使珠海成为区域经济的增长中心、辐射中心和示范窗口，走生态文明的发展道路，努力建设既有活力、又有实力、更有魅力的珠三角现代化区域中心城市。

　　积极落实《纲要》实施方案。要把落实《纲要》实施工作方案作为今年的头等大事。落实责任分工，建立长效机制，加强组织领导、统筹协调和检查评估，围绕“科学发展、先行先试”的要求积极展开探索，用足用活《纲要》的各项政策措施。尽快修编城市发展规划，启动新一轮城市总体规划修编工作，尽快调整完善城市功能布局、空间规划和土地利用总体规划，确保我市各项规划与省相关规划衔接。优化城镇体系和产业布局，大力发展先进制造业和生产性服务业，做大做强主导产业。加快建设高栏港工业区、海洋工程装备制造基地、航空产业园区和国际商务休闲旅游度假区。创新帮扶方式，推进产业和劳动力“双转移”。

　　积极融入珠三角区域一体化。要以世界眼光、战略思维，跳出珠海谋划珠海、发展珠海，主动融入珠三角区域一体化的发展中去。按照省的统一部署，以交通基础设施一体化为切入点，创新合作机制，积极参与构建城市规划统筹协调、基础设施共建共享、产业发展合作共赢、公共事务协作管理的一体化发展格局。以城际轨道交通、广珠铁路、高速公路和管道天然气、电网等为突破口，加快交通和能源基础设施一体化步伐。开展气象、应急等领域合作，协同构建区域环境监测预警体系，加强跨区域水资源保护和利用，加快融入珠三角区域一体化进程。

　　二是以重大项目建设为重点，着力推进经济平稳较快发展。

　　大力推进政府投资计划落实。政府投资是重大基础设施项目建设的重要保障，也是有效应对当前严峻经济形势、保持经济平稳较快发展的有力举措。今年政府投资的重点是重大基础设施建设、民生工程和关键领域等方面。要按基建程序要求，抓紧做好项目可行性研究和评估论证、征地拆迁等前期工作，加快推进项目建设。要抓住资金筹措和项目管理两个关键，积极争取国家和省扩大内需项目资金支持，加快落实银行贷款、追收土地出让欠款，发挥国有企业融资平台作用，积极吸引民间投资，千方百计筹措资金，确保项目资金及时足额到位。要健全机制，加强协调，上下联动，紧密配合，为项目建设创造良好环境。

　　大力推进交通设施建设。建设大交通、推动大发展。建设珠江口西岸交通枢纽城市是《纲要》的要求，也是解决长期制约珠海发展瓶颈问题的关键所在。要开展交通枢纽城市综合体系规划，抓紧编制广珠城际轨道至珠海机场延长线和金港快速路、香海路、黄茅海大桥等重大交通项目规划方案，完善市内快速干道交通规划，形成方便快捷、现代化的海陆空立体交通网络规划体系，为开展新一轮大规模交通建设创造良好条件。要继续推进广珠铁路、广珠城际轨道、机场高速、高栏港高速、凤凰山隧道等项目加快建设，完成湖心路口至白蕉段省道工程改造。新开工建设珠海大道辅道改造项目、井岸二桥、金凤路和翠屏路等交通工程。落实公交优先，新建一批候车亭，加快拱北等交通枢纽站场建设，切实做好拱北口岸、城际轨道、港珠澳大桥三大节点交通对接。要加快完善海空“双港”集疏运体系，加大推进高栏港区基础设施配套力度，发挥好“双港”在珠海经济发展中的引擎作用。完成珠海港总体规划修编，抓紧万山大型深水中转港研究，加快编制荷包岛海洋工程装备区规划，建成第二个5万吨级集装箱泊位，开工建设高栏港区10万吨级主航道、10万吨级煤码头、4个10万吨级集装箱码头等港口工程，加快推进南水作业区大突堤围堰和5万吨级燃气码头项目。

　　大力推进重大项目建设。重大项目是我市经济社会发展的重要动力，必须倾注全力、抢抓机遇、加快建设。要想方设法，争取国家和省批准一批、开工建设一批、建成使用一批、积极储备一批重大项目。科学论证和推进高栏装备制造区与大杧岛的陆岛连接线工程、荷包岛与大杧岛防波堤规划建设，积极推进中船海洋工程装备制造项目。推动航空产业园建设，加快建设中航通用飞机制造基地，申报设立国家级航空产业园，培育和发展航空产业。推动深水海洋工程装备、南海天然气终端、黄茅岛和高栏液化天然气、巨人网络和金山软件总部等项目加快建设。促进壳牌润滑油、丽珠制药等项目建成投产。大力推进500千伏国安输变电工程等电网工程和珠海电厂5号机组建设。

　　大力搞活流通扩大消费。消费是拉动经济增长的重要因素。要落实有效措施，促进城乡居民增加收入，不断提高城乡居民消费能力。要大力引进国际品牌连锁企业投资，推进大型购物中心、商贸物流中心等项目建设，构建大贸易网络，推动商贸流通业加快发展。开展“浪漫之城·生态珠海”旅游年系列活动，办好海岛旅游节、水乡旅游文化节、沙滩音乐节以及国际海钓和龙舟邀请赛，丰富澳门环岛游、圆明新园、农科奇观、西部田园风光和渔家乐等旅游项目和产品内涵，增强海岛观光、生态旅游、休闲度假、商务会展等特色旅游产品吸引力。加快海泉湾二期、海岛和游艇公用码头、游艇俱乐部等项目建设。要新建1-2家五星级以上酒店，提高珠海城市旅游度假接待能力，争取成为我省实施国民旅游休闲计划首批试点城市。抓紧第八届中国航展相关工作，积极筹办一批专业展会。加大保障性住房建设力度，促进房地产市场健康发展。落实“家电下乡”等消费政策，扩大农村地区消费。

　　三是以结构调整为重点，着力推进现代产业体系建设。

　　强化产业规划和政策引导。完善现代产业发展规划。坚持先进制造业与现代服务业“双轮驱动”发展，积极推进重化工业基地、高新技术产业基地、现代服务业基地、生态农业基地建设。继续实施“东部大转型、西部大开发”战略，打造“香洲服务”和“西部制造”品牌。细化产业发展政策，修订《珠海市产业发展导向目录》，鼓励发展用工少占地少、有研发有品牌、高技术高效益的产业项目。大力发展物流、会展、电子商务、服务外包等现代服务业。

　　强化园区集群化发展。按照布局集中、产业集聚、土地集约、环保优先的原则，充分发挥园区主导产业的带动作用，着力培育“八大产业集群”，打造特色园区、品牌园区和效益园区。加快建设以临港重化、海洋工程装备制造和现代港口物流业为主的高栏港经济区，以高新技术、文化创意和高端服务业为主的高新技术产业开发区，以航空制造和空港物流业为主的航空产业园，以电子信息、家用电器和临港加工业为主的富山工业园。做强南屏、三灶、新青科技园和平沙游艇与休闲旅游区，大力发展电子信息、打印耗材、生物医药、游艇和休闲旅游等特色产业。

　　大力发展海洋经济。贯彻落实全省第六次海洋工作会议精神，编制完善海岸、海岛保护与利用规划和海洋功能区划，搞好海洋综合开发利用，加强海岛基础设施建设，推进科技兴海，重点发展海洋运输业、海岛中转仓储业、海洋新兴产业、海洋高新技术产业，大力开发新兴高端滨海旅游产品。抓住省赋予珠海围（填）海造地先行先试权的机遇，结合我市未来城市发展和项目用海要求，按照科学可行的原则开展围海造地，为我市城市发展创造更大空间。

　　四是以内外源经济协调发展为重点，着力提高开放型经济水平。

　　加大力度开展招商引资。招商引资是我市经济工作的重要任务，也是建设现代产业体系的有力举措。要创新招商方式和投资促进机制，保住存量、力扩增量，采取引进内资与外资并举、引进新项目与鼓励增资扩产并举、项目招商与产业招商并举、扩大数量与提高质量并举、外出推介与网上招商并举等措施，大力推动招商引资工作上新水平。要充分利用国内国外两个市场、两种资源，主动承接符合珠海发展规划的产业转移项目。瞄准中央企业开展重大项目招商，瞄准欧美、日韩等发达国家开展先进制造业招商，瞄准港澳开展现代服务业招商，瞄准民营优质企业开展对内招商，瞄准重点产业开展产业招商。积极吸引中央企业、跨国公司、大型民营企业到我市设立地区总部、研发中心和采购中心，大力发展总部经济。

　　加大力度促进外经贸稳定增长。促进外经贸稳定增长是我市实现保增长目标的重要措施，重点是抓好扩大生产、稳定进出口、促进内销等工作。采取政策倾斜、资金倾斜、服务倾斜等措施，鼓励生产性企业壮大生产规模。千方百计落实保持外贸稳定发展的措施，优化出口结构，重点鼓励有自主知识产权的高新技术和机电产品出口，大力拓展新兴市场。千方百计鼓励创立自主品牌，促进加工贸易企业转型升级。千方百计鼓励外商投资和加工贸易企业扩大内销，拓展国内市场。千方百计鼓励有条件的企业“走出去”，支持企业参与国际、国家和行业标准制定活动，提高企业竞争力。

　　加大力度发展中小企业和民营经济。民营经济是我市经济的重要组成部分。要认真落实扶持民营经济和中小企业发展的政策措施，努力营造民营企业家在社会上有地位、政治上有荣誉、经济上有实惠的良好氛围。发挥民营经济发展联席会议作用，及时协调解决发展中的问题。实施“中小企业成长工程”，优先支持有市场、有效益、有发展前景的中小企业转型升级，促进中小企业从“小、散、弱”向集群发展。完善信用担保体系建设，改善中小企业融资环境。加强技术创新、检验检测、人才培训、信息咨询等服务，促进中小企业加快发展。

　　加大力度开展企业服务。服务企业是政府的重要职责。要把强化企业服务作为优化营商环境的重要举措，增强企业克服困难、加大投资、谋求发展的信心。严格执行《珠海市企业和企业经营者权益保护办法》，完善企业办事和投诉平台，健全服务协调机制，提高服务企业能力。进一步落实减轻企业负担各项措施，多管齐下，努力降低企业发展成本。加强经济运行监测和预警工作，及时为企业排忧解难。今年安排企业发展扶持资金5亿元，重点在融资上市、开拓市场、科技研发、品牌建设等方面加大扶持力度。

　　五是以横琴新区规划建设为重点，着力深化珠港澳台合作。

　　规划建设横琴新区。横琴新区是珠港澳合作的重要平台。要完善横琴规划功能布局，整合土地资源，拓展发展空间。完善基础设施，推进项目招商，尽快启动横琴新区建设。要大胆推进横琴新区体制创新、科技创新、合作创新和环境创新，推动区域创新平台、物流服务基地、旅游共同市场建设，努力把横琴新区建设成为发展现代服务业的先行区、粤港澳合作机制的创新区和珠江口西岸城市的服务区。

　　深化珠港澳台经贸合作。加大落实内地与港澳更紧密经贸关系安排力度，做好对港澳开放的先行先试工作。加强产业合作，承接港澳现代服务业的转移和辐射，支持我市港澳加工贸易企业转型升级。推动珠澳跨境工业区向珠澳跨境合作区转型升级，搭建与港澳服务业、高新技术产业合作的新平台。加快建设金湾台湾农民创业园，依托我市现有台资企业，扩大对台经贸合作。

　　深化珠港澳项目合作。项目合作是珠港澳合作的重要内容。特别是随着港珠澳大桥的规划建设，推进重大基础设施对接尤为必要。要抓紧调整相关规划，切实做好交通路网等方面的衔接。加快推进拱北口岸改扩建和横琴口岸相关设施建设，加强跨区域口岸合作，提高大通关效率，发展口岸经济。深化机场等项目的合作，全力推进机场临时口岸、5万吨级集装箱码头和中化格力公用码头对外开放。加快竹银水源工程建设，确保珠澳供水安全。

　　六是以自主创新为重点，着力提高城市核心竞争力。

　　强化企业自主创新地位。实施自主创新行动计划，完善自主创新政策体系。加大科技投入，今年财政预算安排科学技术支出3亿元，增长27.4％，支持企业研发、技术改造、引进国家重点实验室和贷款贴息。引导企业加大研发投入，建立多元化投入机制。落实企业研发费用税前扣除等优惠政策，扶持创业投资和产业投资基金发展，吸引优质创业资本、项目、技术集聚，加快建立以企业为主体、市场为导向、产学研相结合的科技创新体系。

　　加强创新平台建设。平台建设是创新的基础。要发挥大学园区高校智力优势和科技支撑作用，加快大学科技园建设。大力实施知识产权战略、技术标准战略和以质取胜战略。加大对企业技术中心、工程中心、公共实验室和产学研战略联盟的扶持力度，争取组建市级工程中心2-3家，省级工程中心1-2家，电子信息、生物医药、海洋科技、新材料等领域的产学研基地2-3家。加快国家印刷及办公自动化消耗材料质量监督检验中心等项目建设。提高信息化水平，促进自主创新。实施引才优才计划。人才资源是科技进步和推动创新的第一资源。要大力推动科研工作机制创新，加快留学人员创业园和博士后流动站建设，提高对海内外创新型人才和高层次人才的吸引力。实施高端人才和高技能人才培养工程，继续推进广东科技人才（珠海）基地、高技能人才实训（珠海）基地建设。加强创新文化建设，营造鼓励创业、创新、创造的良好社会环境。

　　七是以环境保护为重点，着力加快宜居城市建设。

　　提高城市规划、建设和管理水平。以规划为龙头，按照东西两大板块、组团式发展的思路，优化城市布局。尽快编制完成中心城区控制性详细规划，加快编制西部中心城区概念规划、起步区控制性详细规划。抓紧研究情侣路、湾仔十字门商务区规划。加快推进前山河综合整治工程，积极推进旧城区、城中旧村和旧工业区改造，完成一批横街小巷改造、路灯设置和“水浸黑点”整治等市政工程。加快启动西部城区建设，继续推进西湖城区和温泉新城规划建设，加快井岸城区建设步伐。实施交通畅通工程。坚持建管结合，加快“数字城管”建设。依法拆除违法建筑，规范广告牌设置，清理乱摆卖行为，实施城乡清洁工程，努力实现香洲城区看不见“乱搭建”、看不见“乱摆卖”、看不见“乱丢垃圾”，不断提高城市管理水平。

　　抓好国家环保模范城市和园林城市复查迎检工作。加强环保基础设施建设，是生态文明建设的重要基础。要严格实施《珠海市环境保护条例》。建成医疗垃圾焚烧厂和西坑尾垃圾渗滤液处理厂等设施，加快推进垃圾发电厂技改项目、茶冷迳垃圾填埋场等环保设施建设。确保完成拱北污水处理厂扩建和中心镇污水处理厂的建设，加大污染源在线监控工程和环境应急能力建设力度，加快排洪渠综合整治、污泥处置资源综合利用等迎检重点工程的建设进度，确保我市环保模范城市和园林城市通过国家复查。

　　抓好节能减排工作。节能减排是一项硬任务，必须千方百计确保完成。大力实施生态建设“四个百分百”行动方案，做好循环经济和发展低碳经济试点工作，倡导清洁生产。加大节能减排的监测、统计和考核力度，建立能耗评估制度。加强单位生产总值能耗水平监测，推动重点耗能企业整体搬迁，淘汰污染严重的企业。加快建设企业脱硫等工程，加大海域、内河水域等环境污染治理和监管力度，强化节能减排责任。

　　抓好生态文明建设试点。建设生态文明新特区是我市的重大使命。要科学编制和严格实施环境保护规划，继续开展生态文明创建活动，积极构建节约能源资源和保护生态环境的产业结构、增长方式、消费模式。加强土地执法监察，严厉打击乱挖山取土等违法违规行为，防治地质灾害，落实土地管理共同责任制度。推行节约集约用地，提高土地利用效益。落实全民绿化运动实施方案，推进“双十百绿”工程建设。建设城市森林公园，实施林分林相改造工程，积极申建黄杨山国家森林公园，申报设立竹洲省级水松湿地保护区，推进淇澳红树林生态园建设。扩大城市绿化面积，实施道路绿化、美化工程，逐步形成立体生态景观，提高绿化水平。

　　八是以农村改革发展为重点，着力推进城乡一体化。

　　加快推进农村和农场改革。推进农村土地管理制度改革，以转包、出租、互换、转让、股份合作等多种形式流转土地承包经营权，发展多种形式的适度规模经营，支持农村发展物业出租等集体经济，多渠道增加农民收入。落实最严格的耕地保护制度，进一步完善基本农田、水资源保护补偿机制。继续开展农业政策性保险试点，完善农村金融服务体系。加强农村管理，开展农村社区建设实验工作。深化农村综合改革，培育农民新型合作组织，完善责权利相统一的镇街村居管理体制。加快华侨农场体制改革。

　　加快现代农业发展。推动都市型农业、生态型农业的发展，提高农业产业化、集约化、市场化发展水平。调整农业结构，加快斗门北部生态农业区等现代农业园区建设，推进农产品加工区和物流展销区建设。提高农民生产经营组织化程度，培育壮大农业龙头企业。支持农业龙头企业开展技术研发、基地建设、品牌建设，发展订单农业，带动农业增效、农民增收。加快农产品质量检验检测体系建设，发展无公害绿色农业。加强农业科技推广，推进农业机械化、标准化，改革基层农业技术推广体系。落实强农惠农政策，确保农业专项资金到位，加大农业补贴和被征地农民的扶持力度。

　　加快发展农村公共事业。贯彻落实《关于统筹城乡发展加快推进城乡一体化的实施意见》，加大城乡统筹力度，着力推进城乡发展规划、产业布局、基础设施、公共服务一体化。加大对农村基础设施、水改工程和社会事业的资金投入，引导更多社会资金投向农村。加强标准农田建设，加快城乡防灾减灾工程尤其是西部地区水利设施建设，尽快修复被强台风损毁的水利和市政设施。继续抓好农村公路和危桥改造，加大农村和海岛交通、水电、清洁能源、垃圾等设施建设力度。加快平沙、红旗华侨农场和白藤农场危房改造。

　　九是以改善民生为重点，着力推进各项社会事业全面发展。

　　加快文教体卫事业发展。加快野狸岛歌剧院、珠海博物馆新馆、文化馆等文化设施建设。做好全国文明城市创建工作。筹办首届金秋艺术节。深化文化体制改革，促进文化产业发展。加强文化遗产保护，做好古镇、古村落和名人故居保护开发工作，挖掘和丰富城市文化内涵。继续提升大学园区办学层次和水平。积极创建广东省推进教育现代化先进区和珠海职教基地。推进北师大（珠海）附中二期工程等项目建设。大力发展职业教育，推进市第二职校、市技师学校（技师学院）和市城职院建设。促进民办教育健康发展，探索多元化办学模式。推进镇中心幼儿园建设，规范幼儿园管理。进一步提高教育水平，促进城乡教育均衡发展。开展全民健身活动，办好各类体育赛事，积极备战省运会和全运会。完成121个行政村的体育设施建设。加快实施基本医疗保障制度，理顺镇村卫生医疗机构体制和财政供给机制，完善农村卫生服务中心功能，加大薄弱卫生院改造和区级医疗卫生机构建设力度，扎实推进市人民医院北区、市疾控中心、市第二中医院等项目建设，提高医疗服务水平和公共卫生应急保障能力。

　　强化就业和社会保障工作。坚持劳动者自主择业、市场调节就业、政府促进就业方针，实施更加积极的就业政策，多渠道开发就业岗位。优化创业环境，鼓励自主创业，以创业带动就业。统筹城乡就业，加强劳动保障和人力资源服务平台建设，加大农村劳动力就业转移培训力度，加强高校毕业生就业工作。公益性岗位优先安排就业困难人员。实施“双百工程”，推动城乡适龄青年百分百接受技工教育、百分百实现技能就业。进一步扩大社会保险覆盖面，重点解决好非就业群体社会保障问题。研究探索城乡居民基本养老保险制度，建立基本医疗保险门诊费用统筹机制。完善最低生活保障制度和城乡医疗救助制度，加大对低收入群体的帮扶力度，切实做到应保尽保。加大劳动保障监察执法力度，加强农民工工作，切实维护劳动者的合法权益。

　　强化食品药品安全、产品质量和安全生产监管。坚持安全发展，夯实和谐社会建设基础。切实抓好食品药品质量安全和生产安全，实施最严格的食品药品和安全生产标准，建立权责利相对应的法律追究惩治体制。建立食品安全评估机制，实行全程监督管理。加强产品质量监管，落实打假工作责任制，严厉打击各种违法违规生产经营行为。进一步落实安全生产责任制，扎实开展安全生产宣教、执法、治理“三项行动”，健全市、区、镇（街）安全生产监管体系。

　　强化维稳、综治、信访和应急工作。认真履行维护稳定第一责任，健全维稳工作机制，注重从源头上预防和化解矛盾，切实把社会矛盾纠纷解决在基层、解决在当地、解决在萌芽状态。构建适应社会转型的利益协调、诉求表达和权益保障机制，健全人民调解队伍和基层调解组织，加强法律援助工作，积极化解、妥善处理人民内部矛盾。加强信访工作，健全维稳评估机制和处置工作机制，妥善处理改革发展过程中遇到的热点、难点问题。大力推进社会治安综合治理，完善社会治安防控体系，严密防范、严厉打击敌对势力的破坏渗透活动和各类违法犯罪活动，做好反恐工作，进一步提高群众安全感。加大森林防火力度。加强应急管理体系建设，完善市、区、镇（街）和企业应急救援联动机制，完善海上搜救应急机制，提高我市应急救援能力，确保人民群众生命财产安全。

　　大力发展其它社会事业。支持中央和省驻珠单位以及各人民团体充分履行职责。加强双拥共建、国防建设和民兵预备役工作。加强计生服务和管理，稳定低生育水平。做好科普工作，提高市民科学素质。加快发展妇女儿童、红十字、慈善和残疾人事业，继续做好民族宗教、外事、侨务、档案、人防、仲裁、打私、海防、气象、地震、地质、地方志、港澳流动渔民和对口援建帮扶工作。

　　十是以增收节支为重点，着力提高科学理财水平。

　　力争财税收入稳定增长。积极推进社会综合治税体系建设，加大收入征管力度，提高税费征管科学化、专业化、精细化水平，确保应收尽收。强化纳税服务，引导单位和个人依法纳税。积极培育税源，强化非税收入征管，挖掘增收潜力。加强财税运行分析，健全异动预警机制。

　　优化财政支出结构。按照保运转、保重点、保民生和集中财力办大事的原则，严格控制预算外支出，优化财政资源配置。珍惜纳税人的劳动成果，用好纳税人每一分钱。加大教育、医疗、社保、就业、环保、文体、农林水事务等方面支出，真正做到“取之于民、用之于民”。加大财政专项资金和政府投资项目的跟踪审计，加强财政支出绩效评估，完善财政监督管理机制。

　　创新财政管理体制。按照财权与事权相匹配的原则，完善财政体制，调动各级政府发展经济和科学理财的积极性。建立收入征收权、财力分配权、资金使用权、财政管理权相互制约机制。增强预算编制的科学性和准确性，加强预算管理和监督，增强预算透明度。要完善重大建设项目稽察制度和绩效评估制度，强化预算监督，提高政府投资效益。

　　十一是以管理体制改革为重点，着力推进体制机制创新。

　　积极推进社会管理体制改革。推进公共服务市场化改革，推广政府向社会组织购买服务制度，推进以特许经营方式为主的公用事业改革，建立多元化的公共服务供给模式。完善社区治理模式，壮大社会工作者队伍，支持自愿服务体系建设。大力发展社会组织，简化社会组织登记注册程序，培育和扶持重点行业组织、公益慈善组织、社区社会组织。继续开展示范街道和“六好”平安和谐社区创建活动。强化实有人口综合规范管理，积极探索建立流动人口服务管理“一证通”制度。

　　积极推进行政管理体制改革。以建设服务型政府为目标，加快转变政府职能，全面推进政企、政资、政事、政府与市场中介组织“四个分开”。开展机构改革，加快建立权责一致、分工合理、决策科学、执行畅顺、监督有力、高效运行的行政管理新体制。开展第四轮行政审批制度改革，规范行政审批行为，依法下放行政审批事项，健全行政许可听证、行政公示、否定事项报备等制度，推行审批管理“零收费”制度，非行政许可事项压缩一半。充分发挥行政效能投诉中心的作用，加大行政审批电子监察力度。

　　积极推进重点领域改革。继续发挥市场在资源配置中的基础性作用。加快金融领域改革，加强金融生态建设，防范和化解金融风险，促进金融产业发展。继续深化国有企业改革，整合国有企业资源，加快国有经济结构调整步伐。完善法人治理结构和出资人制度、国有资本经营预算制度。建立健全规范有效的国有企业董事会和监事会，完善外部董事和外派监事制度，实现国有企业决策权、监督权和执行权分开。建立健全国有企业评价体系、风险管理体系和约束激励机制。推进价格和收费改革。积极申报综合保税区和保税港区，优化生产服务和港口物流环境。稳妥推进农贸市场管理体制改革，改善市民购物环境。

　　十二是以效能政府建设为重点，着力加快服务型政府建设步伐。

　　加强民主法制建设。深入学习贯彻国务院《全面推进依法行政实施纲要》和《关于加强市县人民政府依法行政的决定》，提高政府工作人员依法办事的自觉性。加强行政执法监督，促进严格执法、公正执法和文明执法。做好市人大议案、代表建议和政协提案的办理工作。认真听取各民主党派、工商联和无党派人士的意见。成立专家顾问委员会，进一步提高重大决策水平。健全政务公开制度，扩大公民有序政治参与，切实保障人民群众的知情权、参与权、表达权和监督权。发展基层民主，完善村（居）民自治制度。

　　加大监督力度。监督是规范政府行为、推进依法行政的重要保障。坚持向市人大及其常委会报告工作，自觉接受监督。主动接受市政协的监督，接受纪检监察和司法机关的监督。注重发挥新闻舆论和社会监督作用。重视人民群众通过行政复议、行政诉讼等法定渠道，加强对政府机关及其工作人员的监督。要向社会公开政府重大决策及其落实情况、公开政府部门职责、公开政府审批事项、公开政务信息和依法公开审计结果。加强政府系统内部监督，支持监察、审计部门依法独立履行监督职责，尤其要加强政府投资项目资金使用、工程招标投标、土地出让开发、财政转移支付、政府采购和国有资产转让等方面的监督，确保政府运作阳光透明、廉洁高效。

　　提高行政效能。开展“落实年”活动，全力推动各项工作全面落实。采取切实有效措施，进一步明确职责、优化流程、强化问责，促进行政效能的提高。充分发挥部门职能，理顺市、区、镇（街）各级的职责分工，依法扩大基层自主权。减少环节、缩短时限，做到急事急办、特事特办，着力解决“办事难”问题。全面推广“一站式”服务和“网上审批”，建立和完善跨部门统一互联的电子政务平台，不断提高政府服务社会能力。杜绝有令不行、有禁不止行为，严格执行行政首长问责、行政过错责任追究等规章制度，做到有责必问，不断提升政府执行力和公信力。正确处理规范与效率的关系，营造干事创业的良好环境。

　　加强政风建设。政府组成人员必须率先垂范、严格自律，当好公仆。加强行风评议，深入开展创建“群众满意基层站所”活动。大力弘扬艰苦奋斗精神，牢固树立过紧日子的思想。厉行节约，坚持勤俭办一切事业，确保公务购车和用车经费、会议经费、公务接待费用、机关出国（境）费用、办公经费预算零增长，坚决制止奢侈浪费。深入开展廉政建设和反腐败斗争，坚决纠正各种不正之风。切实加强公务员队伍的教育、管理和监督，进一步提升整体素质，努力建设一支素质高、能力强、作风好的公务员队伍。

　　各位代表，今年是新中国成立60周年，又是实施《纲要》的第一年。任务繁重，使命光荣。让我们在省委、省政府和市委的坚强领导下，以拼搏的精神、以创新的勇气、以务实的作风，团结带领全市人民，努力拼搏，奋发有为，为建设生态文明新特区、争创科学发展示范市而不懈奋斗！
